# Does the social credit system construction reduce enterprises’ overinvestment? quasi-natural experimental evidence from China

**DOI:** 10.1371/journal.pone.0318328

**Published:** 2025-02-07

**Authors:** Guozhen Zhang, Yurui Xia, Yiting Xie, Yuanxiang Dong

**Affiliations:** School of Economics and Management, Taiyuan University of Technology, Taiyuan, China; Middle Tennessee State University Jennings A Jones College of Business, UNITED STATES OF AMERICA

## Abstract

As an important part of improving the socialist market economic system, the social credit system focuses on the market’s micro behavior, which emphasizes the credit self-discipline of enterprises in financial management and checks the orientation for enterprises’ investment decisions. This study selected Shanghai and Shenzhen A-share listed companies for 2012–2023 as samples and used the pilot reform of China’s social credit system as a quasi-natural experiment to test the impact and mechanisms of the construction of the social credit system on enterprises’ overinvestment. The main findings show that the social credit system construction alleviates enterprises’ overinvestment behaviors, realized by the inhibiting effect of internal controls, the reverse effect of risk monitoring, and the guiding effect of business environment optimization. Heterogeneity analyses illustrate that the social credit system construction has a more obvious inhibitory effect on overinvestment for enterprises that are less subject to financing constraints, have higher internet penetration, have better financial development levels, are eastern, non-manufacturing, and large-scale. The conclusions provide new perspectives and references for the government and enterprises to re-examine the economic effects of the social credit system construction, which can promote capital usage efficiency and guide enterprises toward rational management.

## Introduction

As one of the “troikas” driving economic growth, investment is critical for sustainable and healthy development. Effective investment is a prerequisite for the survival and development of micro-entities in the market, and it plays an essential role in enterprises’ profitability and growth ability. Maintaining a reasonable and moderate investment is conducive to expanding the production scale and coping with operational risks. However, driven by the principal-agent problem of modern enterprise systems and sufficient internal cash flow, managers make inefficient investment decisions regarding overinvestment to build a “manager empire”, that is, accepting investments with a net present value (NPV) less than 0. The economic consequences of overinvestment mainly stem from the principal-agent relationship and financial risk, embodied in the following two aspects. It intensifies the conflicts of interest between managers, shareholders, investors, and enterprises. It also causes high bankruptcy costs, high risk of capital loss, and ease of falling into financial distress. Therefore, enhancing financial soundness and enterprise value has increased academic attention to overinvestment.

“Opinions on Promoting High-quality Development of the Social Credit System and Promoting the Formation of a New Development Pattern” highlight the necessity to strengthen market credit constraints, establish and improve the risk prevention and resolution mechanisms of “early detection, warning, and disposal”, and support financial institutions in using big data technology to strengthen tracking, monitoring and early warning. The “Outline of the Social Credit Systems Construction Plan (2014–2020)” focuses on four primary areas: government, business integrity, social integrity, and judicial credibility. Among them, improving business integrity is the key, emphasizing that enterprises should be guided to strengthen credit self-discipline in production, operation, financial and labor management. Based on relatively perfect laws and regulations and guaranteed by the government’s robust supervision system, the social credit system has established and improved the credit information-sharing mechanism, formed an efficient and smooth national economic cycle, and constructed an external governance system for new development patterns [1]. Enterprises act as credit providers in the investment process and credit demanders in the financing process. If there is a lack of credit, providers are afraid to invest, and demanders find it challenging to obtain production and operating funds, leading to inefficient investment, financing, and business activities that would not be conducive to realizing long-term development goals. Therefore, the social credit system provides a “gatekeeper orientation” towards enterprise management, financing, investment, and other decisions.

The construction of the social credit system focuses on the market’s micro behaviors and risk control. Under the dual role of external “implicit” supervision and internal “explicit” governance, enterprises dare not break their trust, which restrains opportunistic behavior of overinvestment. In addition, the social credit system is a basic requirement and important support for the business environment, as well as a guide for enterprises to invest efficiently. Therefore, the relationship between the construction of the social credit system and overinvestment is worthy of discussion. This study takes the pilot reform of China’s social credit system as a quasi-natural experiment, A-share listed companies in Shanghai and Shenzhen for 2012–2023 as samples, and examines the influence of the social credit system construction on enterprise overinvestment. We find that the social credit system construction alleviates overinvestment, realized by the inhibiting effect of internal controls, the reverse effect of risk monitoring, and the guiding effect of business environment optimization. Further analyses show that the social credit system construction has a more obvious inhibitory effect on overinvestment for enterprises that are less subject to financing constraints, have higher internet penetration, have better financial development levels, are eastern, non-manufacturing, and large-scale.

The main marginal contributions are as follows: First, most studies on factors influencing external constraints on overinvestment are based on financial perspectives, such as the bank joint credit system and short-selling mechanism, while few studies focus on non-financial factors. From a non-financial perspective of the social credit system, this study examines its influence on enterprises’ overinvestment. It expands the understanding of the economic effect of the social credit system construction. Second, this study opens the black box for overinvestment. Based on the multi-angle anatomy of the influence mechanisms of the social credit system on overinvestment, it verifies relevant paths for strengthening internal controls, risk monitoring, and optimizing the business environment. It provides direct evidence for improving enterprises’ investment efficiency and enriching the research on relevant influencing factors and internal mechanisms. Finally, these conclusions significantly guide enterprises to optimize investment decisions and promote efficient capital usage in rational management. Meanwhile, the conclusions have profound policy implications. The social credit system has a positive policy-oriented effect on the economic behaviors of micro-enterprises and provides a new perspective for credit improvement.

## Literature review

The existing literature on overinvestment mainly holds three viewpoints: (1) The trait theory of managers. This view focuses on exploring the psychological characteristics of managers’ overconfidence [2,3], power expansion [4,5], and background characteristics [6–10] that drive overinvestment. (2) Market competition theory. There are two diametrically opposing views on the effect of market competition on overinvestment. Market competition restrains management’s self-interested behavior by reducing overinvestment and alleviating information asymmetry between managers and external stakeholders [11]. However, enterprises intensify overinvestment driven by “seizing the first opportunity” and “keeping up with the Joneses” under market competition [12,13]. (3) Government intervention theory. Faced with promotion tournaments and economic growth targets, local governments have the incentive to intervene in enterprises’ investment to aggravate the overinvestment tendency [14–16].

Based on these three viewpoints, academics believe that overinvestment can be alleviated through internal governance and external constraints. From the internal governance effect, most studies show that internal controls have a negative regulatory effect on overinvestment [17–19]. Considering the external constraint effect, existing literature mainly focuses on bank joint credit [20], debt financing constraints [21], analyst tracking [22], institutional investors [23], and government supervision [24]. These external entities restrain overinvestment.

The impact of the social credit system can be discussed from the perspectives of the government, enterprises, and individual economic entities. First, the decline in social credit would lead to an increase in the scale of hidden debt and financing costs of local governments [25]. Second, the social credit system can regulate social behaviors, and “reward and punish” measures related to the credit system might become a primary method for the government to deal with dishonesty [26]. Third, some literature examines the impact of the social credit environment on the growth ability of enterprises [27,28], financialization tendency [29], profit uncertainty [30], innovation [31], stock price crash risk [32], IPO overflow [33] and foreign subsidiaries’ performance [34]. Fourth, besides sincerity, trust, and other policy initiatives to effectively regulate individual behaviors, the social credit system involves personal data acquisition and full disclosure of credit information [35]. Social dishonest environments inhibit household entrepreneurship [36] and encourage participation in private lending markets [37]. However, a transparent environment under the social credit system may infringe on citizens’ privacy and political freedom.

In summary, the literature concerning external environmental constraints to overinvestment focuses more on the financial level; few studies focus on the non-financial level. Few studies have explored the impact of the social credit environment on enterprises’ investment, focusing only on investment, such as financialization, without efficiency. Therefore, it is necessary to study the influence of the social credit system on overinvestment from a non-financial perspective.

## Theoretical analysis and research hypotheses

### The social credit system construction and overinvestment

The modern market economy is essentially a credit economy, and credit is an essential factor affecting enterprise capital. Social credit is becoming more critical for judging how well economic agents perform and ensuring that a market economy works well [38]. With the support of the social credit system, the capital market can identify enterprises’ investment efficiency and development potential more accurately, strengthening ongoing supervision and evaluation from stakeholders and the public. In addition, the social credit system relies on the credit information system covering the whole society to realize the effective link between enterprises and various entities [39], helping well-developed enterprises secure additional financial support. Therefore, managers are encouraged to focus on the cost of improper investment and the value of efficient investment. The possibility of investing in poor projects based on opportunistic motives is reduced to alleviate overinvestment from managers’ self-interest [40].

The construction of the social credit system requires sound information disclosure. Improving the market’s complex and opaque trading conditions helps enterprises break through information barriers and inefficient resource allocation problems. It automatically eliminates potential risk projects with significant problems by optimizing the market mechanism, reducing the possibility of incorrect investments in risk projects (NPV<0) due to information asymmetry, and guiding enterprises to make more valuable investments. Based on this, we propose the following hypothesis:

***H1*.** The social credit system construction can significantly reduce enterprises’ overinvestment.

### Internal control mechanism: Inhibition effect

The external governance environment can affect governance structure, and by improving the internal governance, enterprises’ inefficient investments can be restricted. As a crucial external governance environment, the social credit system aims to establish stricter enterprise governance requirements by improving information transparency [41]. Under the guidance of the social credit system, the control level of enterprise’s monetary funds and the effectiveness of shareholder supervision have improved.

From the control activities perspective, the technology and data of the social credit system have entered a fast lane [26], and the legislation based on this has become an inevitable requirement to improve China’s legal construction. As of April 2022, 50 laws and 59 administrative regulations provide special credit provisions, and 19 localities issue provincial social credit-related local regulations. Bringing the social credit system into strict rule of law requires continuous improvement in internal control. As the specific carrier and core link of internal controls, control activities have a monetary fund management system. The high-quality cash flow authorization, approval, and management processes ensure optimal cash holding by limiting managers’ autonomy [42], thus restricting the tendency to overinvest.

From the supervision perspective, once an enterprise has dishonest behavior caused by negative situations such as performance decline or a capital chain break due to overinvestment, the social credit system plays a deterrent role in the joint punishment system and places such enterprises on the management blocklist, making it difficult to move. The damaging publicity of enterprises’ financing, operations, and investment activities directly threatens shareholders’ profits, thus stimulating them to supervise managers. This kind of checks and balances, formed by improving regulatory motivation, alleviates the principal-agent problem, inhibits managers’ opportunistic behaviors, and reduces overinvestment [43,44].

In summary, the social credit system improves enterprises’ governance, enhances internal control quality, and exerts a restraining effect of internal control mechanisms on overinvestment. Incomplete internal control would aggravate overinvestment [45], whereas complete internal control encourages managers to make reasonable and reliable investment decisions. Therefore, the inhibitory effect of the social credit system on overinvestment relies on internal control components. Based on this, we propose the following hypothesis:

***H2*.** The social credit system construction reduces enterprises’ overinvestment through the inhibitory effect of the internal control mechanism.

### Risk monitoring mechanism: Forced effect

The social credit system exposes credit information, such as enterprise registration, license approval, annual reports, and administrative punishment. Such credit records cover enterprises and financial institutions, which can broaden the channels for them to control credit risks and improve the efficiency of risk monitoring through incentives for integrity and punishments for dishonesty [46]. For example, “Guiding Opinions on Accelerating the Social Credit System Construction and Building a New Credit-based Supervision Mechanism” points out that credit risk identification would penetrate all aspects of enterprise production and operation through a new credit supervision mechanism that connects the entire supervision link. In addition, “Opinions on Promoting a High-quality Social Credit System Construction and Promoting the New Development Pattern Formation” supports financial institutions in using big data and other technologies to strengthen tracking, monitoring, and warning.

Compared to the punishment mechanism for dishonesty, the social credit system can empower financial institutions to identify enterprise credit, depending more on the prescreening mechanism [47]. Improving the prescreening mechanism is an essential measure for the social credit system construction. Banks and other financial institutions identify the quality level and risk degree of financing enterprises according to information publicized by the social credit information system and improve the accuracy of financial institutions in predicting the enterprise solvency and preventing the default risk of investment projects. In the social credit system construction, improving the prescreening mechanism is conducive to strengthening credit risk assessment and monitoring of enterprises by banks, making information exchange between banks and enterprises realize Pareto improvement. Banks’ increasingly efficient risk monitoring mechanism requires managers to consider future financing channels, with limited financing sources and increasing business process uncertainty. Therefore, constructing a social credit system enhances financial institutions’ ability to identify and monitor enterprises’ risk. It forces managers to reduce their risk tolerance through risk-monitoring mechanisms and adopt conservative investment strategies, that is, to give up high-risk investment projects and reduce overinvestment [48]. Based on this, we propose the following hypothesis:

***H3*.** The social credit system construction reduces enterprises’ overinvestment through the forced effect of the risk-monitoring mechanism.

### Business environment optimization mechanism: Guiding effect

As an informal regulation, social credit is a behavioral pattern formed unconsciously and spontaneously by social subjects. However, the business environment is a formal regulation, a form of conscious creation. The New Institutional Economics School believes that informal regulation is the precursor of formal regulation. The business environment is the external ecological environment that enterprises rely on for survival and development [49]. Based on this, we believe that, as an important part of a good business environment, the complete social credit system should play a leading role in optimizing the business environment.

Rational investors can make effective decisions only after analyzing relevant information, including accounting information, but the market situation is still inefficient and is closer to semi-strong. The social credit system construction is committed to consolidating the responsibility of enterprises’ information disclosure to improve market transparency. Since the 18th National Congress of the CPC, China has actively practiced the full coverage of unified social credit codes and launched measures such as credit red and blocklists and credit easy loans. These initiatives aim to achieve information disclosure and application to improve the business environment and enhance market effectiveness. Thus, investors can obtain the correct information and filter incorrect information in an efficient market, improving investment efficiency.

In addition, an excellent social credit environment helps improve resource allocation efficiency to promote the flow of surplus resources from low efficiency to high efficiency and finally transfer to the highest value [50]. It provides new ideas for business environment improvement, reflected in the reform of “delegating management services” at all levels of government to reduce ineffective resource waste, such as streamlining approval and licensing, implementing tax and fee reductions, and optimizing business processes. These measures mitigate unnecessary institutional costs and significantly improve operational efficiency so that enterprises have a keen insight into investment projects, improving the investment quality and enhancing return on investment decisions [51]. Based on this, the fourth hypothesis is proposed.

***H4*.** The social credit system construction reduces enterprises’ overinvestment through the guiding effect of the business environment optimization.

## Research design

### Sample selection and data source

This study uses Shanghai and Shenzhen A-share listed companies for 2012–2023 as samples. The data are obtained from the National Bureau of Statistics, China Government Network, China Executive Information Disclosure Network, China City Statistical Yearbook, and the CSMAR, EPS, CNRDS, and DIB databases (China Stock Market & Accounting Research Database, Emerging Patents Statistics Database, China National Research Data Services Database, DIB Internal Control, and Risk Management Database) and are manually compared. The data are processed as follows. First, the samples of listed financial, ST, and *ST companies are excluded (The complete terms are “Special Treatment” and “Special Treatment with a Star” companies, referring to listed companies that have severe issues with their financial or operational conditions). This approach is based on the significant differences in business models, risk management, and regulatory environments between the financial sector and other industries, leading to distinctive statistical characteristics in financial enterprise data. Simultaneously, enterprises such as ST and *ST often face financial difficulties or operating problems that may lead to lower reliability and accuracy in their financial reports. Moreover, the abnormal financial indicators of these enterprises may affect the accuracy of the research findings. Therefore, to ensure the comparability and representativeness of the study’s conclusions, we exclude these samples. Second, the samples with missing data were eliminated. Then, samples with negative absolute values of the residuals calculated by Ordinary Least Squares (OLS) regression using Richardson’s investment efficiency model are removed. The estimated negative residual value represents underinvestment samples. However, our study focuses on samples with overinvestment, indicated by the positive absolute value of the residual. Finally, continuous variables are winsorized by 1% and 99% quantiles to avoid the influence of data outliers. A total of 12155 observations are obtained for 2012–2023.

### Variable definition

#### Explained variable

Reference to [52], Model (1) was constructed and calculated. The positive residual was used to measure overinvestment (*OINV*), and the negative residual was used to measure underinvestment. This study retains the samples with positive residuals (multiply the value by 100). The smaller the residual value, the higher the investment efficiency.


$$\begin{array}{l} INV_{it}=\delta_{0}+\delta_{1}INV_{it-1}+\delta_{2}GROWTH_{it-1}+\delta_{3}LEV_{it-1}+\delta_{4}CASH_{it-1}+\delta_{5}AGE_{it-1}+\delta_{6}SIZE_{it-1}\\ \;+\delta_{7}RETURN_{it-1}+YEAR_{t}+IND_{i}+\varepsilon_{it} \end{array}$$ (1)

Where *INV* is enterprises’ investment expenditure, *GROWTH* is growth level, *LEV* is asset-liability ratio, *CASH* is cash holding, *AGE* is listing years of enterprises, *SIZE* is enterprise size, *RETURN* is stock return rate, *YEAR* and *IND* represent the year and industry dummy variable. To make the regression results more visually presented on an appropriate scale, we expand the data on overinvestment by 100 times.

### Explanatory variable

The social credit system construction is a comprehensive project aimed at improving the overall credit level of society. It collects and evaluates the credit information of individuals and enterprises, establishes credit files, incentivizes trustworthy behavior, and penalizes dishonest behavior through the credit scoring system. The system was first implemented in some demonstration cities and demonstration zones and gradually extended to the whole country through the practice and exploration in these areas. The aim was to promote the improvement of the social credit system and the sound development of the economic order. The specific construction of the social credit system is shown in Table 1.

**Table 1:** The social credit system construction city.

Year	Type	City
2015	Pilot city	Shenyang, Nanjing, Wuxi, Suqian, Hangzhou, Wenzhou, Yiwu, Hefei, Wuhu, Qingdao, Chengdu
2016		Beijing, Shanghai, Hohhot, Wuhai, Dalian, Anshan, Liaoyang, Suifenhe, Suzhou, Taizhou, Anqing, Huaibei, Xiamen, Fuzhou, Putian, Weifang, Weihai, Dezhou, Rongcheng, Zhengzhou, Nanyang, Wuhan, Xianning, Yichang, Huangshi, Guangzhou, Shenzhen, Zhuhai, Shantou, Huizhou, Luzhou
2018	Demonstr-ation zone	Nanjing, Suzhou, Suqian, Hangzhou, Wenzhou, Yiwu, Xiamen, Weihai, Weifang, Rongchen, Huizhou, Chengdu
2019		Shanghai, Anshan, Wuxi, Hefei, Huaibei, Wuhu, Anqing, Fuzhou, Putian, Qingdao, Zhengzhou, Wuhan, Xianning, Yichang
2021		Tianjin, Chongqing, Xingtai, Dalian, Yingkou, Siping, Changzhou, Huai’an, Yangzhou, Kunshan, Ningbo, Huzhou, Jinhua, Quzhou, Zhoushan, Taizhou, Lishui, Jinan, Yantai, Jining, Dezhou, Xintai, Luohe, Nanyang, Jingmen, Guangzhou, Shenzhen, Foshan, Baoshan, Yan’an
2023		Shijiazhuang, Qinhuangdao, Tangshan, Renqiu, Huanghua, Qian’an, Luanzhou, Shahe, Nangong, Zunhua, Dingzhou, Wuhai, Songyuan, Baicheng, hou, Nantong, Yancheng, Taizhou, Jiangyin, Rugao, Jiaxing, Shaoxing, Jiande, Jiangshan, Yuyao, Suzhou, Bengbu, Zhangzhou, Quanzhou, Pingxiang, Zibo, Zaozhuang, Rizhao, Liaocheng, Longkou, Zhucheng, Tengzhou, Anyang, Hebi, Xinxiang, Jiaozuo, Xinyang, Changsha, Chenzhou, Yongzhou, Liuyang, Dongguan, Zhongshan, Nanning, Liuzhou, Haikou, Mianyang, Guangyuan, Yulin, Lanzhou, Jinchang, Zhangye, Yinchuan

*Source*: Data compiled by the authors.

According to the different times when the city where the enterprise is located enters the pilot reform of the social credit system, this study sets dummy variable *TREAT* to make a better causal judgment. If the city where the enterprise is located is included in the pilot reform of the social credit system, the value is 1; Otherwise, it is 0. Then, set the time dummy variable *POST*, that is, when the year in which the enterprise is located became a social credit pilot city, and in subsequent years, the value is 1; Otherwise, it is 0.

#### Mediating variables

Mediating variables are internal control quality (*IC*), risk level (*UNCER*), and business environment (*BER*). Based on the existing research, *IC* is calculated by the internal controls Index divided by 100 from the DIB database.

The stock price volatility is used as a proxy indicator of enterprise risk level (*UNCER*). The standard deviation of a stock’s daily return is used to measure its volatility over a year. Daily returns are calculated as follows:

$$lgLEV_{itd}=\log(1+LEV_{itd})$$ (2)

Among them, *LEV*_*itd*_ represents the actual daily return rate of the stock *i* on the day *d* of the year *t*. *lgLEV*_*itd*_ represents the logarithm of the corresponding daily stock return.


$$Mean_{it}=\sum_{d=1}^{n}lgLEV_{itd}/n$$ (3)

$$UNCER_{it}=\sqrt{\sum_{d=1}^{n}{\left(lgLEV_{itd}-Mean_{it}\right)}^{2}}$$ (4)

In the above formula, *UNCER*_*it*_ represents the annual stock price volatility of the stock *i* in year *t*.

The index of business environment (*BER*) is derived from the Blue book of China’s Urban Business Credit Environment Index (*CEI*). The business environment index of each city is constructed from seven dimensions: credit market tools, enterprise credit management functions, credit information system construction, government credit supervision, credit improvement in key areas, credit education and enterprises. The *BER* can be used to reflect the comprehensive improvement effect of the social credit system construction in various cities on the business environment.

#### Control variables

This study selects a set of control variables that are used in previous studies, which includes property right nature (*SOE*, 1 for state-owned enterprises and 0 for non-state-owned enterprises), listing age (*AGE*, the year when the enterprise is established), asset-liability ratio (*LEV*, total liabilities/total assets), revenue growth rate (*GROWTH*, (amount of operating income in current period—amount of operating income in previous period)/ amount of operating income in previous period), cash flow level (*CFO*, (monetary funds + trading financial assets)/total assets), ownership concentration (*OC*, number of shares held by top 10 shareholders/total number of shares), duality (*DUAL*, when the chairman and the general manager are the same, it is 1; otherwise, it is 0), independent director ratio (*INDEP*, number of independent directors/total number of director), equity balance degree (*BALANCE*, sum of shares held by the second to five major shareholders/shares held by the first major shareholder), and executive gender (*SEX*, 1 for male executives and 0 for female executives).

#### Model specifications

This study estimates difference-in-difference with multiple time periods in Models (5) to (7), which incorporate firm fixed effects, year fixed effects, industry fixed effects, and cluster standard errors at the firm level:

$$OINV_{it}=\alpha_{0}+\alpha_{1}TREAT\times POST_{it}+\sum_{k}\chi_{k}CONTROLS_{kit}+YEAR_{t}+IND_{i}+\varepsilon_{it}$$ (5)

$$MEDIATOR_{im}=\beta_{0}+\beta_{1}TREAT\times POST_{it}+\sum_{k}\delta_{k}CONTROLS_{kit}+YEAR_{t}+IND_{i}+\varepsilon_{it}$$ (6)

$$OINV_{it}=\gamma_{0}+\gamma_{1}TREAT\times POST_{it}+\gamma_{2}MEDIATOR_{im}+\sum_{k}\eta_{k}CONTROLS_{kit}+YEAR_{t}+IND_{i}+\varepsilon_{it}$$ (7)

Where O*INV*_*it*_ represents overinvestment in year t of enterprise i; *TREAT×POST*_*it*_ is the dummy variable of the social credit system construction; *CONTROLS* are a series of control variables, and k is the number of control variables. In addition, the year and industry fixed effects are controlled. *ε*_*it*_ is a random disturbance term.

**Fig 1:**
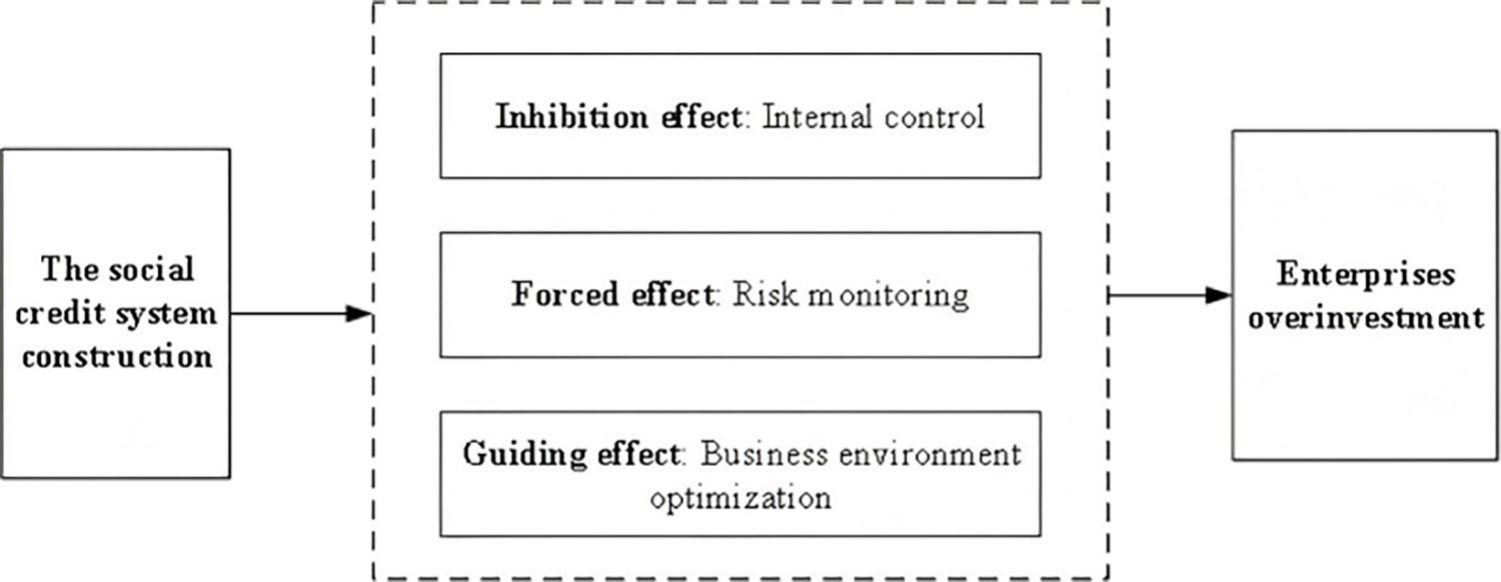
Theoretical analysis framework.

Meanwhile, this study combines a three-step method to build Model (6) and Model (7) based on Model (5) to test the inhibiting effect of internal controls, the forcing effect of risk monitoring, and the guiding effect of business environment optimization. Among them, *MEDIATOR*_*im*_ is the mediating variables, which include *IC*, *UNCER*, and *BER* respectively (Fig 1 shows the theoretical analysis framework).

If the coefficient of *TREAT×POST* (α_1_) is significantly negative in the Model (5), then hypothesis H1 is supported; that is, the construction of the social credit system can significantly reduce enterprises’ overinvestment. On the basis that the coefficient α_1_ in the Model (5) is significantly negative, the mediating mechanisms are further tested. If β_1_ is significantly positive, γ_1_ is significantly negative, and the absolute value of γ_1_ is less than the absolute value of α_1_, this would support the hypothesis that *IC* is the mediating variable (H2 is proved). The same idea applies to the *BER* mediating variable (hypothesis H4). If both β_1_ and γ_1_ are significantly negative, and the absolute value of γ_1_ is less than the absolute value of α_1_, this would support the hypothesis of *UNCER* as a mediating variable (H3 is proved).

## Empirical analysis

### Descriptive statistics

Table 2 presents descriptive statistics. The mean of *OINV* is 3.535, the standard deviation of *OINV* is 3.796, the minimum is 0.029, and the maximum is 18.267, indicating that the degree of overinvestment of enterprises varies greatly. The mean of *TREAT×POST* is 0.402, indicating that in the sample, about 40.2% of enterprises’ office addresses in pilot reform of the social credit system. *IC* ranges from 3.444 to 9.954, with little difference between the mean and median, indicating that the distribution of internal controls is uniform. The mean of *UNCER* is 1.249, and the standard deviation is 0.722, meaning that there is little difference. *BER* is between 62.801 and 90.630, and both the mean and median are small, showing that the business environment needs to be further optimized. In addition, the descriptive statistical results of control variables are basically consistent with previous relevant research.

**Table 2:** Descriptive analysis.

Variables	Observations	Mean	Min	P50	Max	SD
*OINV*	12155	3.535	0.029	2.199	18.267	3.796
*TREAT×POST*	12155	0.402	0.000	0.000	1.000	0.490
*IC*	12116	6.539	3.444	6.674	9.954	0.946
*UNCER*	12155	1.249	0.214	1.089	3.680	0.722
*BER*	11011	76.681	62.801	76.115	90.630	5.982
*SOE*	12155	0.311	0.000	0.000	1.000	0.463
*AGE*	12155	2.156	0.693	2.303	3.466	0.802
*LEV*	12155	0.421	0.059	0.417	0.866	0.196
*GROWTH*	12155	0.186	-0.509	0.127	2.184	0.369
*CFO*	12155	0.196	0.018	0.162	0.636	0.131
*OC*	12155	57.999	23.166	58.760	89.837	15.067
*DUAL*	12155	0.313	0.000	0.000	1.000	0.464
*INDEP*	12155	0.376	0.182	0.364	0.800	0.055
*BALANCE*	12155	0.762	0.000	0.606	4.000	0.615
*SEX*	12155	0.949	0.000	1.000	1.000	0.220

*Note*: *N* = 12155, there are a few missing values for the mediating variables *IC* and *BER*.

### Correlation test

Table 3 presents the correlation coefficients among the variables. It can be seen that there is a significant negative correlation coefficient between *TREAT×POST* and *OINV*; in addition, there is a specific correlation between most variables. This correlation provides preliminary evidence for our understanding of the relationship between variables and lays the foundation for further regression analysis. At the same time, the correlation coefficients between most variables are lower than 0.3 (especially the mediating variables *IC*, *UNCER*, and *BER*), indicating that although there is a certain degree of correlation, the relationships between variables are not strong, thus reducing the possible problems caused by multicollinearity to a certain extent.

**Table 3:** Correlation analysis.

***Panel A***								
**Variables**	***OINV***	***TREAT×*** ***POST***	***SOE***	***AGE***	***LEV***	***GROWTH***	***CFO***	
*OINV*	1							
*TREAT×POST*	-0.057***	1						
*SOE*	-0.113***	-0.072***	1					
*AGE*	-0.155***	-0.010	0.468***	1				
*LEV*	-0.134***	0.0003	0.288***	0.385***	1			
*GROWTH*	0.029***	0.009	-0.077***	-0.086***	0.035***	1		
*CFO*	0.101***	0.064***	-0.154***	-0.297***	-0.421***	0.008	1	
*OC*	0.055***	0.024***	-0.020**	-0.348***	-0.089***	0.079***	0.132***	
*DUAL*	0.058***	0.076***	-0.308***	-0.255***	-0.128***	0.021**	0.096***	
*INDEP*	0.018**	0.072***	-0.077***	-0.026***	-0.0004	-0.005	0.009	
*BALANCE*	0.023**	0.065***	-0.228***	-0.156***	-0.121***	0.045***	0.067***	
*SEX*	-0.019**	-0.030***	0.064***	0.022**	0.023**	0.0004	-0.036***	
*IC*	0.003	0.025***	-0.060***	0.071***	0.023**	-0.162***	-0.055***	
*UNCER*	0.080***	-0.051***	-0.187***	-0.134***	-0.032***	0.092***	0.064***	
*BER*	-0.041***	0.418***	0.029***	-0.032***	0.006	-0.009	0.101***	
***Panel B***								
**Variables**	***OC***	***DUAL***	***INDEP***	***BALANCE***	***SEX***	***IC***	***UNCER***	***BER***
*OC*	1							
*DUAL*	0.032***	1						
*INDEP*	0.031***	0.107***	1					
*BALANCE*	0.009	0.050***	-0.029***	1				
*SEX*	-0.008	-0.006	-0.025***	0.010	1			
*IC*	-0.144***	0.013	-0.005	0.047***	-0.013	1		
*UNCER*	-0.034***	0.111***	0.047***	0.083***	-0.022**	-0.112	1	
*BER*	0.040***	0.032***	0.046***	0.014	-0.028***	0.055***	0.002	1

Note

***, **, and * indicate significance at the 1%, 5%, and 10% levels, respectively.

### Benchmark regression analysis

Column (1) in Table 4 is the result that only explained variables are added into the model, and Column (2) controls fixed effects of year and industry on the basis of Column (1). The coefficients of *TREAT×POST* are -0.967 and -0.897, which are significantly negative at the 1% level. It is preliminarily verified that there is a significant negative correlation between the social credit system construction and overinvestment. Control variables are added to Column (3), and the coefficient of *TREAT×POST* is -0.357, which is significant at the 5% level; Column (4) not only adds control variables but also controls the fixed effects, and the coefficient of *TREAT×POST* is -0.368, and it is significant at the level of 1%. During the process of data curation, to make the regression results more visually presented on an appropriate scale, we expanded the data on overinvestment by 100 times. This approach is common in economic research and aims to make the economic significance of the coefficients more apparent. Therefore, the obtained regression coefficient is actually 100 times that before the original unadjusted scale. Therefore, the obtained regression coefficient is actually 100 times that before the original unadjusted scale, which means that when we observe a coefficient of -0.368 in Column (4), it actually represents an impact ratio of -0.368%, not -36.8%. The social credit system construction can suppress the overinvestment of the enterprise by 0.368% at the average level. This verified H1.

**Table 4:** Benchmark regression results.

Variables	(1)	(2)	(3)	(4)
	*OINV*	*OINV*	*OINV*	*OINV*
*TREAT×POST*	-0.967***	-0.897***	-0.357**	-0.368***
	(-8.055)	(-7.199)	(-2.573)	(-2.604)
*SOE*			-0.381	-0.183
			(-1.273)	(-0.623)
*AGE*			-0.465***	-0.490***
			(-3.032)	(-3.046)
*LEV*			-2.356***	-2.040***
			(-4.660)	(-3.930)
*GROWTH*			0.027	0.001
			(0.243)	(0.013)
*CFO*			4.956***	5.185***
			(9.242)	(9.432)
*OC*			0.007	0.006
			(0.959)	(0.895)
*DUAL*			0.083	0.065
			(0.595)	(0.463)
*INDEP*			-0.577	-0.691
			(-0.496)	(-0.592)
*BALANCE*			-0.573***	-0.640***
			(-3.558)	(-3.959)
*SEX*			-0.202	-0.059
			(-0.616)	(-0.180)
*IND FE/YEAR FE*	No	Yes	No	Yes
*INDIVIDUAL FE*	Yes	Yes	Yes	Yes
*CLUSTER*	Yes	Yes	Yes	Yes
*CONSTANT*	3.925***	17.784***	5.239***	18.176***
	(81.200)	(5.487)	(6.291)	(5.732)
OBSERVATIONS	12155	12155	12155	12155
ADJ_R^2^	0.009	0.036	0.044	0.067

*Note*: T values are in parentheses, and

***, **, and * indicate significance at the 1%, 5%, and 10% levels, respectively.

### Mechanism tests

The role of Column (6) and Column (1) in Table 5 is identical; they effectively represent a re-estimation of the benchmark model (i.e., Model 5) to ensure comparability of results. Although the same model is used, the number of observations is different. As can be seen from Table 5, Column (1) is the result of a re-benchmark regression to ensure that the same sample of *IC* without missing values is used in a three-step process. In Column (2), the coefficient of *TREAT×POST* is 0.128, which is significant at a 1% statistical level, indicating that the social credit system construction can improve the internal control quality of enterprises by 12.8% on the average level. According to Column (3), the coefficient of *TREAT×POST* is still significantly negative, and the absolute value decreases from 0.369 to 0.353 compared with when *IC* is not added, indicating that *IC* plays a part of mediating effect, which demonstrates H2.

**Table 5:** Mechanism tests results.

Variables	(1)	(2)	(3)	(4)	(5)	(6)	(7)	(8)
	*OINV*	*IC*	*OINV*	*UNCER*	*OINV*	*OINV*	*BER*	*OINV*
*TREAT×POST*	-0.369***	0.128***	-0.353**	-0.305***	-0.300**	-0.363**	0.989***	-0.290**
	(-2.612)	(3.858)	(-2.496)	(-11.692)	(-2.107)	(-2.509)	(9.739)	(-1.988)
*IC*			-0.129***					
			(-2.863)					
*UNCER*					0.224***			
					(3.575)			
*BER*								-0.074***
								(-3.403)
*CONTROLS*	Yes	Yes	Yes	Yes	Yes	Yes	Yes	Yes
*IND FE/ YEAR FE*	Yes	Yes	Yes	Yes	Yes	Yes	Yes	Yes
*INDIVIDUAL FE*	Yes	Yes	Yes	Yes	Yes	Yes	Yes	Yes
*CLUSTER*	Yes	Yes	Yes	Yes	Yes	Yes	Yes	Yes
*CONSTANT*	18.222***	6.367***	17.399***	0.093	18.155***	17.542***	72.912***	22.907***
	(5.750)	(11.944)	(5.533)	(0.375)	(5.732)	(5.480)	(40.538)	(6.525)
OBSERVATIONS	12116	12116	12116	12155	12155	11011	11011	11011
ADJ_R^2^	0.067	0.083	0.068	0.053	0.069	0.069	0.073	0.070

*Note*: T values are in parentheses, and

***, **, and * indicate significance at the 1%, 5%, and 10% levels, respectively.

In Column (4), the coefficient of *TREAT×POST* is -0.305, which is significant at the 1% level, indicating that the social credit system construction can enhance the risk monitoring mechanism by 30.5% on the average level, that is, it reduces the risk level of enterprises. According to Column (5), after both *TREAT×POST* and *UNCER* were included in the model, the coefficient of *TREAT×POST* is significantly negative, but the absolute value decreases from 0.368 to 0.300 compared with when *UNCER* is not added, indicating that *UNCER* plays a part of the mediating effect, and H3 is supported.

Column (6) is the result of re-benchmark regression in the sample, ensuring that there are no missing *BER* values. In Column (7), the coefficient of *TREAT×POST* is 0.989, which is significant at the 1% level, indicating that the social credit system construction can exert its guiding effect and optimize the business environment by 98.9% on the average level. According to Column (8), after both *TREAT×POST* and *BER* were included in the model, the coefficient of *TREAT×POST* is significantly negative, and the absolute value decreased from 0.363 to 0.290 compared with that without *BER*. It shows that *BER* plays a part of the mediating effect, and H4 is supported.

### Robustness tests

#### Parallel trend test

Progressive DID requires that experimental and control groups maintain the same trends before policy implementation. Different cities are included in the pilot reform of the social credit system at different times; it is not possible to set a time dummy variable as the critical point of an event, but a dummy variable for the relative time value of each city is included in the pilot reform of the social credit system. Referring to [53], Model (8) was constructed to conduct a parallel trend test.


$$\begin{array}{l} OINV_{it}=\lambda_{0}+\lambda_{1}Before4_{it}++\lambda_{2}Before3_{it}+\lambda_{3}Before2_{it}+\lambda_{4}Before1_{it}+\lambda_{5}Current_{it}+\lambda_{6}After1_{it}\\ +\lambda_{7}After2_{it}+\sum_{k}\omega_{k}CONTROLS_{kit}+YEAR_{t}+IND_{i}+\varepsilon_{it} \end{array}$$ (8)

**Fig 2:**
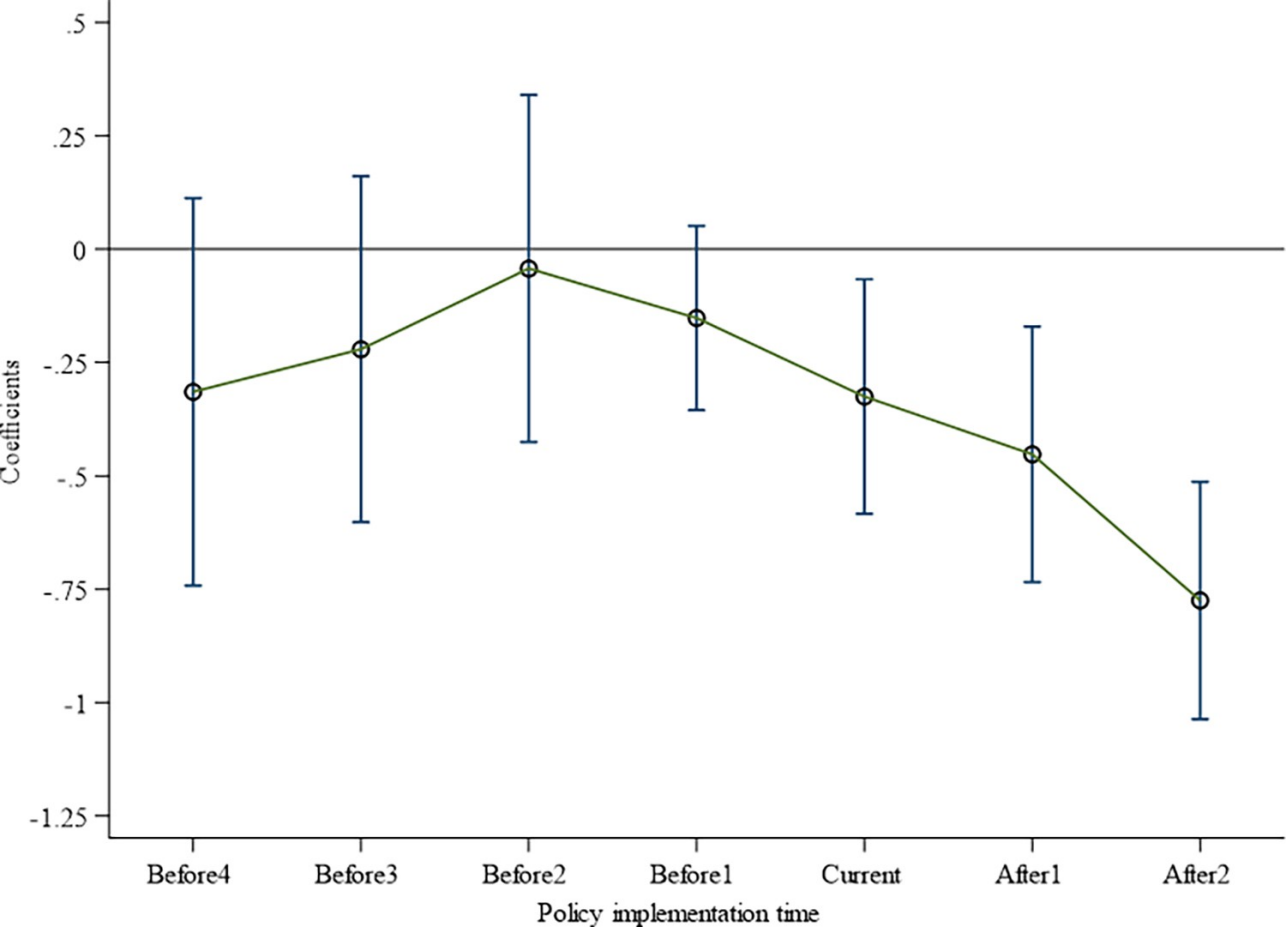
Parallel trend test results.

Where the time dummy variable is the observed value of the first n years, the current year, and the last n years of the social credit system construction, and the value is zero without the social credit system construction. This study introduces a series of relative year dummy variables (Before4, Before3, Before2, Before1, Current, After1, and After2) to examine the dynamic characteristics of enterprise overinvestment. If other unobserved characteristics influence overinvestment, it should not show a significant difference in time for cities undergoing social credit system reforms. However, if enterprises’ overinvestment is serious and social credit system reform is carried out, the overinvestment of local enterprises and enterprises in other regions should differ. Column (1) in Table 6 shows that the coefficients of Before4, Before3, Before2, and Before1 were not significant, indicating that before the social credit system construction, there was no significant difference between the experimental and control groups, which satisfied the parallel trend hypothesis. Fig 2 is generated within a 90% confidence interval, and there is no significant difference in the time trend of overinvestment between affected and unaffected enterprises. In addition, after the pilot reform of the social credit system, the overinvestment of affected enterprises was significantly reduced, which is consistent with the benchmark conclusions.

**Table 6:** Robustness tests.

Variables	(1)	(2)	(3)	(4)	(5)	(6)
	Parallel trend test	PSM-DID	PSM-DID	Clustering to the city level	Alternative explanatory variable	Add missing variables
	*OINV*	*OINV*	*OINV*	*OINV*	*OINV*	*OINV*
*Before4*	-0.285					
	(-1.099)					
*Before3*	-0.202					
	(-0.873)					
*Before2*	-0.0440					
	(-0.192)					
*Before1*	-0.122					
	(-1.006)					
*Current*	-0.295**					
	(-1.998)					
*After1*	-0.759***					
	(-4.966)					
*After2*	-0.884***					
	(-4.269)					
*TREAT×POST*		-0.967***	-0.332**	-0.368**		-0.351**
		(-8.056)	(-2.409)	(-2.456)		(-2.473)
(TREAT×POST*)					0.182***	
					(6.086)	
*CONTROLS*	Yes	No	Yes	Yes	Yes	Yes
*SIZE*						-0.411***
						(-7.007)
*BOARD*						-0.142***
						(-2.778)
*GDP*						0.032***
						(3.397)
*SHARE*						-0.013*
						(-1.831)
*IND FE/YEAR FE*	Yes	No	Yes	Yes	Yes	Yes
*INDIVIDUAL FE*	Yes	Yes	Yes	Yes	Yes	Yes
*CLUSTER*	Yes	Yes	Yes	Yes	Yes	Yes
*CONSTANT*	18.098***	3.925***	2.355	18.173***	24.741***	18.772***
	(5.608)	(81.234)	(0.832)	(5.991)	(7.975)	(6.659)
OBSERVATIONS	12155	12145	12145	12155	7406	12155
ADJ_R^2^	0.068	0.009	0.052	0.067	0.117	0.070

*Note*: T values are in parentheses, and

***, **, and * indicate significance at the 1%, 5%, and 10% levels, respectively.

#### Placebo test

**Fig 3:**
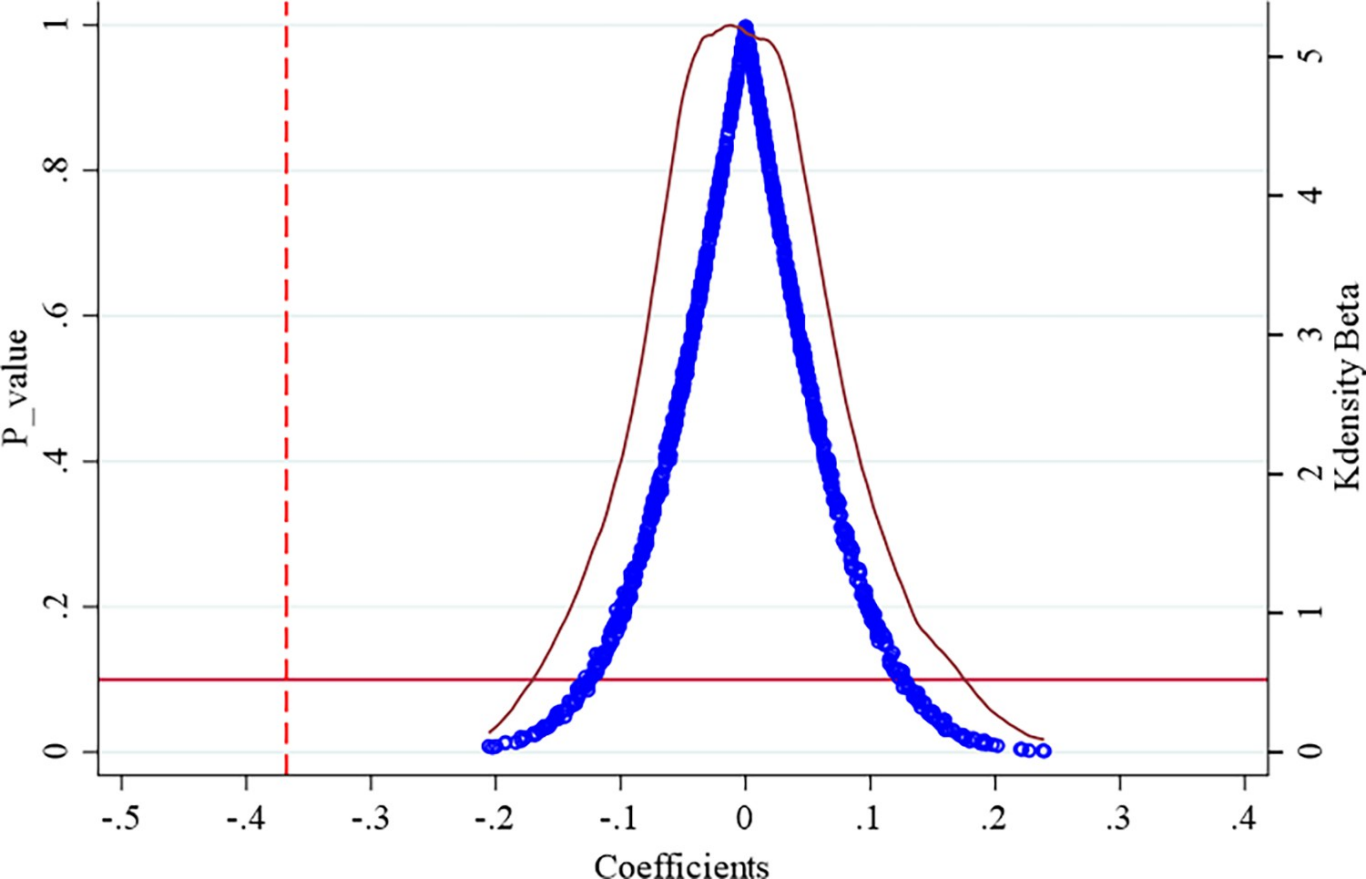
Placebo test results. *Note*: The dashed line is the coefficient from the correct regression, ∘ indicates p_value and — indicates kdensity beta.

This study uses a false pilot year for the social credit system construction to determine whether it reduces overinvestment. Based on existing research [54], we make the pilot year of the social credit system construction random (by computer) and generate a new variable (*credit*). This random process is repeated 1000 times, and after that, the effect of the social credit system construction on reducing overinvestment is no longer significant. Fig 3 shows that the coefficients of *credit* for the random social credit system construction are concentrated around 0, which is much higher than the value of -0.368 obtained by the benchmark estimation. This indicates that the measurement error of the original treatment group is tolerable, and the placebo test is established, which supports this conclusion.

#### PSM-DID

Considering that the limited number of the social credit system construction, including complete samples related to *IC*, *UNCER* and *BER*, the propensity score matching (PSM) method is used to test the robustness of the results to reduce selection bias caused by different initial conditions between the experimental and control groups. Specifically, taking the control variables as matching variables, the kernel matching method is used to assign more weight to control group samples closer to the propensity score of the experimental group samples.

**Fig 4:**
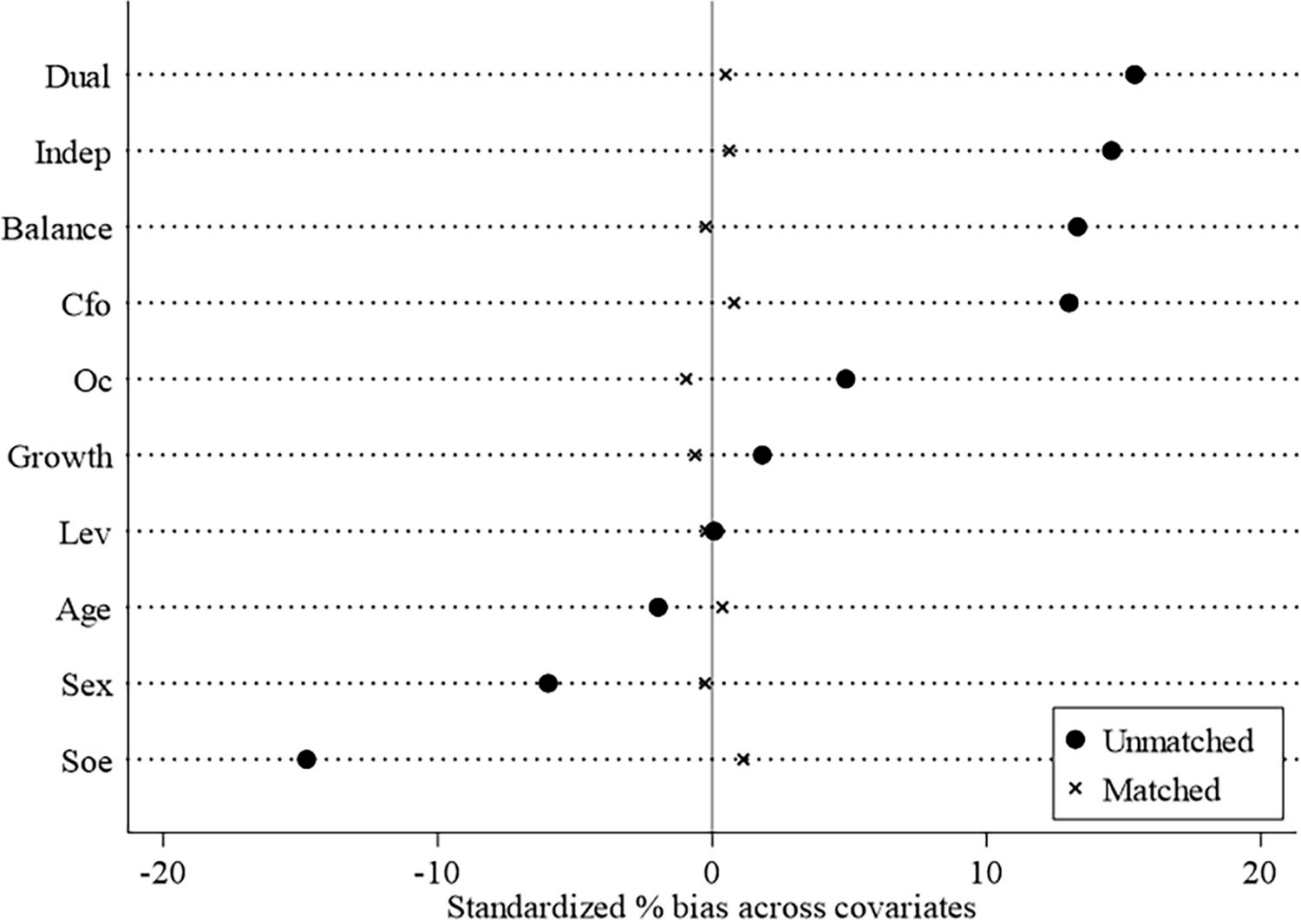
Control variables’ difference between the experimental group and the control group after PSM.

In order to reduce the impact of sample heterogeneity and ensure the accuracy of DID estimation, analyzing the differences before and after sample matching is necessary. As shown in Fig 4, the standard deviations of all variables are significantly reduced after matching; the differences between the variables are reduced. This indicates no significant difference in the variables between the experimental and control groups after matching; the selected matching variables and methods are appropriate. After verifying the rationality of the PSM-DID method, a DID analysis is conducted based on the matching results. Column (2) in Table 6 presents that the estimated coefficient of *TREAT×POST* is still significantly negative when control variables and fixed effects are not added. When these effects are added to Column (3), the coefficient of *TREAT×POST* is negative at the 5% significance level, depicting the same sign and direction of the relationship as earlier regressions.

#### Adjust clustered standard errors

Considering that enterprises in the same city are similar, this study further incorporates clustered standard errors at the city level to alleviate the estimation bias and allow the correlation of residual terms at the city level. As in Column (4) in Table 6, the coefficient of *TREAT×POST* is significantly negative at the 5% level. The research results remain unchanged.

#### Alternative measures of the explanatory variable

In order to avoid manual calculation and measurement errors, the ratio of the number of dishonest enterprises in prefecture-level cities to the gross regional product (TREAT×POST*) is used as a proxy variable for the social credit system construction. As in Column (5) of Table 6, the coefficient of TREAT×POST* is 0.182, significant at the 1% level. That is, the more dishonest enterprises in a city, the more serious the overinvestment of enterprises. The conclusions remain unchanged.

#### Add missing variables

Other missing variables may drive the relationship between the social credit system and enterprise overinvestment. Therefore, more variables are added: gross domestic product (*GDP*) is controlled at the regional level; enterprise scale (*SIZE*, the natural logarithm of an enterprise’s total assets), board size (*BOARD*, the natural logarithm of the number of directors), and the proportion of senior executives’ shares (*SHARE*, the number of shares held by senior executives at the end of the year/total number of shares) is controlled. According to Column (6) in Table 6, the significance level of the coefficient of *TREAT×POST* at 5% is negative. The main conclusions do not change.

### Endogeneity test

This study uses an instrumental variable method to overcome the endogeneity problem. Based on the conditions of strong correlation and weak exogeneity, the number of historical colleges is used as an instrumental variable (*IV*). From the perspective of relevance [25], proposed that the more historical colleges there are, the stronger the Confucian culture will be, and awareness of social credit will improve. Regarding exogenesis, the number of historical colleges is not directly related to overinvestment, meeting the exogenous conditions.

Table 7 shows the results of the two-stage least squares method. The first-stage results show that the coefficient of *IV* is significantly positive at the 1% level, indicating that regions with more historical colleges have a better social credit system. In addition, the F-value of Column (1) is greater than 16.38, indicating that the IV does not have a weak instrumental variable. Column (2) shows the second-stage results. The coefficient of *TREAT×POST* is significantly negative at the 1% level, and the social credit system construction significantly reduces overinvestment. Therefore, after controlling for endogeneity using the instrumental variable method, the regression results remained robust.

**Table 7:** Endogeneity test results.

Variables	(1)	(2)
	*TREAT×POST*	*OINV*
*IV*	0.004***	
	(7.303)	
*TREAT×POST*		-4.529***
		(-2.638)
*CONTROLS*	Yes	Yes
*IND FE/YEAR FE*	Yes	Yes
OBSERVATIONS	11273	11273
R^2^ /F	41.553	0.045

*Note*: T values are in parentheses, and

***, **, and * indicate significance at the 1%, 5%, and 10% levels, respectively.

## Further analysis

### Heterogeneity of financing constraints

When enterprises are subject to strong financing constraints, it is more difficult to obtain external funds; therefore, there may be a preventive incentive to invest. In order to verify the regulatory effect of financing constraints at the enterprise level, this study uses the SA index to measure the degree of financing constraints [55], including only two variables with strong externalities and little change over time: enterprise size and age. According to the mean SA, the samples are divided into strong and weak financing constraint groups, and a grouping regression analysis is performed. The results are shown in Table 8 (*Panel A*).

**Table 8:** Heterogeneity tests results.

***Panel A***						
**Variables**	**(1)**	**(2)**	**(3)**	**(4)**	**(5)**	**(6)**
	**Strong financing constraints**	**Weak financing constraints**	**High internet penetration rate**	**Low internet penetration rate**	**Good financial development**	**Poor financial development**
	***OINV***	***OINV***	***OINV***	***OINV***	***OINV***	***OINV***
*TREAT×POST*	0.051	-0.491**	-0.367*	-0.352	-0.426**	-0.012
	(0.241)	(-2.035)	(-1.844)	(-1.160)	(-2.100)	(-0.050)
*CONTROLS*	Yes	Yes	Yes	Yes	Yes	Yes
*IND/YEAR FE*	Yes	Yes	Yes	Yes	Yes	Yes
*INDIVIDUAL FE*	Yes	Yes	Yes	Yes	Yes	Yes
*CLUSTER*	Yes	Yes	Yes	Yes	Yes	Yes
*CONSTANT*	26.544***	2.448	3.446**	8.834***	5.377***	15.922***
	(10.312)	(0.730)	(2.238)	(3.599)	(2.334)	(6.946)
OBSERVATIONS	6180	5975	5072	7083	5892	6263
ADJ_R^2^	0.080	0.076	0.068	0.090	0.087	0.076
***Panel B***						
**Variables**	**(1)**	**(2)**	**(3)**	**(4)**	**(5)**	**(6)**
	**Eastern**	**Central & Western**	**Manufacturing**	**Non-manufacturing**	**Large-scale**	**Smaller-scale**
	***OINV***	***OINV***	***OINV***	***OINV***	***OINV***	***OINV***
*TREAT×POST*	-0.464***	0.086	-0.309	-0.488**	-0.699**	0.006
	(-2.864)	(0.287)	(-1.630)	(-2.266)	(-2.438)	(0.034)
*CONTROLS*	Yes	Yes	Yes	Yes	Yes	Yes
*IND/YEAR FE*	Yes	Yes	Yes	Yes	Yes	Yes
*INDIVIDUAL FE*	Yes	Yes	Yes	Yes	Yes	Yes
*CLUSTER*	Yes	Yes	Yes	Yes	Yes	Yes
*CONSTANT*	19.026***	7.694***	5.842***	4.286***	0.798	6.793***
	(9.489)	(4.375)	(5.606)	(3.068)	(0.377)	(3.453)
OBSERVATIONS	8642	3513	8135	4020	6076	6079
ADJ_R^2^	0.077	0.072	0.034	0.070	0.069	0.087

*Note*: T values are in parentheses, and

***, **, and * indicate significance at the 1%, 5%, and 10% levels, respectively.

Column (1) shows that the coefficient of *TREAT×POST* is 0.051, and no significant relationship is observed; this coefficient is significantly negative at the 5% level in Column (2). Therefore, the social credit system construction has a more obvious role when the degree of financing constraints is lower. Here, the social credit system construction can reduce 0.491% of enterprises’ overinvestment at the average level.

### Heterogeneity of regional internet penetration

With the popularity of the internet, the transmission of enterprise-related information has become more timely and inexpensive, and information disclosure has become more transparent. Negative news about enterprises causes huge reputation loss, public opinion pressure, and external activity restrictions. The number of internet broadband access users (thousands) in each prefecture-level city is used to measure the internet penetration rate. According to its mean, the sample is divided into high and low regional internet penetration rate groups.

Column (3) of Table 8 (*Panel A*) shows that the coefficient of *TREAT×POST* is significant at the 10% level, while Column (4) shows that the coefficient of *TREAT×POST* is not significant. This illustrates that in areas with high internet penetration, the social credit system construction plays a more obvious role. Here, the social credit system construction can reduce 0.367% of enterprises’ overinvestment at the average level.

### Heterogeneity of regional financial development

Regions with high financial development are rich in resources, efficient in allocation, and mature in various financial policies and regulations, providing favorable support for the improvement of enterprise information disclosure systems and promoting investment efficiency. By referring to existing research [56], this study adopts the ratio of the total balance of deposits and loans of financial institutions in various places at the end of the year to the gross regional product to measure the regional financial development level. Samples larger than the average value are divided into groups with good regional financial development, and samples smaller than the average value are divided into groups with poor regional financial development.

It can be seen that the coefficient of *TREAT×POST* is only significant in Column (5) in Table 8 (*Panel A*), which means that in areas with good financial development levels, the social credit system construction has a more obvious role. Here, the social credit system construction can reduce 0.426% of enterprises’ overinvestment at the average level.

### Heterogeneity of region

Significant differences in economic development, living standards, and resource endowments exist among different regions in China. Compared with the underdeveloped central and western regions, the eastern regions have developed economies and abundant resources, and the construction of a credit system is more accessible to implement. In addition, the eastern region may have a more open and efficient economic environment. Therefore, this study examines whether heterogeneity in the effect of the social credit system construction on enterprises’ overinvestment exists in the eastern, central, and western regions with different economic development levels. For the grouping regression, the samples were divided into two groups according to the registration location of the enterprises (eastern, central, and western regions).

Column (1) in Table 8 (*Panel B*) reports that the coefficient of *TREAT×POST* is significant at the level of 1%; Column (2) presents that the coefficient of *TREAT×POST* is not significant. This illustrates that there is obvious regional heterogeneity in the mitigation effect of the social credit system construction on the enterprises’ overinvestment, which is obvious in the economically developed eastern regions, but it plays no obvious role in the relatively backward central and western regions. In eastern regions, the social credit system construction can reduce 0.464% of enterprises’ overinvestment at the average level.

### Heterogeneity of industry

Industry characteristics may play a heterogeneous role in the practice of the social credit system. Specifically, the restraint and incentive effects of the social credit system are limited due to the capital intensity and production process characteristics of manufacturing enterprises. Non-manufacturing enterprises have greater flexibility and sensitivity to market signals. This gives full play to the positive effects of the credit system, effectively guides rational investment decisions, and prevents overinvestment. Therefore, the samples are divided into manufacturing and non-manufacturing enterprises for grouping regression.

Column (3) in Table 8 (*Panel B*) reports that the coefficient of *TREAT×POST* is not significant; Column (4) presents that the coefficient of *TREAT×POST* is significant. This illustrates that the construction effect of the social credit system in different industries has obvious differences, which has no obvious effect on manufacturing enterprises but has a significant effect on non-manufacturing enterprises. Here, the social credit system construction can reduce 0.488% of enterprises’ overinvestment at the average level.

### Heterogeneity of enterprise size

Differences in enterprise size may also affect the social credit system’s role in alleviating overinvestment. Large enterprises generally receive more public and regulatory attention; thus, their investment behavior is more susceptible to the credit system. In contrast, for small-scale enterprises, the awareness and influence of the social credit system are relatively low. Therefore, the role of the social credit system differs among enterprises of varying sizes. Based on the median enterprise size, the samples are divided into two groups for grouping regression.

Column (5) in Table 8 (*Panel B*) reports that the coefficient of *TREAT×POST* is significant at the level of 5%; Column (6) presents that the coefficient of *TREAT×POST* is not significant. This illustrates that enterprise size plays a heterogeneous role in the relationship between the social credit system and overinvestment, which plays an obvious role in large-scale enterprises but not in smaller-scale enterprises. In large-scale enterprises, the social credit system construction can reduce 0.699% of enterprises’ overinvestment at the average level.

## Research conclusions and policy recommendations

This study analyzes the impact of the social credit system construction on enterprises’ overinvestment and draws the following conclusions: First, the social credit system construction reduces enterprises’ overinvestment; Second, it acts on overinvestment by improving internal control quality, strengthening risk monitoring and optimizing the business environment; Third, the social credit system construction has a more obvious inhibitory effect on enterprises with weak financing constraints, higher internet penetration, better financial development, and eastern, non-manufacturing, and large-scale.

This study provides a conclusion that is different from the dominant finance perspective. Compared with most studies that focus on financial perspectives, such as capital markets and financial instruments, this study starts from the non-financial perspective of the social credit system. Through quasi-natural experiments, it is found that the social credit system construction can significantly reduce enterprises’ overinvestment. By capturing the causal relationship between the two, this study transcends the limitation of previous research conclusions and policy revelations, remaining at the same level. It provides practical guidance on how social governance measures can promote stable and healthy interactions between enterprises and the economic society. Furthermore, this study examines the mechanisms through which the construction of the social credit system influences enterprises’ overinvestment by identifying inhibition, forced, and guiding in aligning overinvestment behaviors. Additionally, it uncovers the differentiated impacts of the social credit system construction across enterprises with varying characteristics and regions at different levels of development. These insights enhance our understanding of how the social credit system shapes enterprises’ investment decisions and offer comprehensive application scenarios for formulating and refining policies tailored to specific enterprise features and regional conditions.

The following policy recommendations are proposed. First, the new credit-based supervision mechanism must be improved to accelerate the pace of the social credit system construction. Governments should encourage more batches of social credit demonstration cities, strengthen the credit commitment system, provide trustworthy incentives and punishment for breach of trust to implement credit supervision, and standardize enterprise investment behaviors. It must give full play to the information disclosure role of the “Credit China” website, the national enterprises’ credit information publicity system, the public institution registration management website, and the platform for social organization credit information publicity. Second, it should grasp the application point of the social credit system to promote enterprises’ investment efficiency. The establishment of strict and sound decision-making standards and risk assessment processes within enterprises can inhibit irrational investment behavior. The government and enterprises should jointly build an early risk-warning system to promote self-risk management within the organization and optimize capital allocation. Improving overall investment efficiency, improving the business environment, and guiding wise investment choices is long-term work. Third, governments continue to improve the internal and external investment environments for enterprises’ investment activities. Financial support combined with various financial instruments can alleviate the financing constraints of enterprises, actively promote the internet, and strengthen the construction of a basic financial credit information database and application of internet technology. Realizing interconnection, interoperability, and mutual sharing of credit information on a wider range of platforms exerts the market value of credit information to a greater extent. It should bring together internal and external forces of enterprises to fully utilize the potential and advantages of the social credit system and guide them to achieve efficient development through efficient investment.

Limitations and suggestions for further studies. The first is the research design. This study uses a quasi-natural experimental method to treat the construction of the social credit system as a binary variable cross-multiplying term. Although some hidden confounding factors can be controlled using the quasi-natural experimental method, it may not fully capture the subtle differences and complexity of the degree of development of the social credit system. Additionally, reducing the social credit system to a single indicator may ignore the interaction of different dimensions and levels within the system, limiting the ability to gain a deeper understanding of the mechanisms that influence overinvestment behavior. Future studies can build more detailed and comprehensive multidimensional evaluation indicators to measure the development and soundness of the social credit system and more accurately assess its impact on overinvestment. More advanced statistical and econometric methods, such as structural equation modeling (SEM), can also be explored to address more complex data relationships and causal inference problems. Second, this study is based mainly on the office addresses of enterprises to determine whether they belong to the scope of a policy pilot. Owing to the complexity of data processing and the research focus, it did not consider how to manage a sample of enterprises with multiple branches or in different cities. This study provides a basis for analyzing the policy effects of the construction of the social credit system at the overall enterprise level. With the continuous maturity of the construction of the social credit system and the improvement in the accessibility of data acquisition, future research can be further refined to the level of branches. In addition, future research on the economic consequences of the social credit system could be extended to other trustworthiness focuses besides overinvestment. Simultaneously, the model is gradually expanded to include many trust indicators and assessment tools so that stakeholders can more accurately assess and understand the impact and value of the social credit system.
